# Microglia in Alzheimer's Disease

**DOI:** 10.1155/2014/437483

**Published:** 2014-08-14

**Authors:** Ying Li, Meng-Shan Tan, Teng Jiang, Lan Tan

**Affiliations:** ^1^Department of Neurology, Qingdao Municipal Hospital, School of Medicine, Qingdao University, No. 5 Donghai Middle Road, Qingdao 266071, China; ^2^Department of Pathology, Qingdao Municipal Hospital, Qingdao 266071, China; ^3^Department of Neurology, Qingdao Municipal Hospital, College of Medicine and Pharmaceutics, Ocean University of China, Qingdao 266003, China; ^4^Department of Neurology, Qingdao Municipal Hospital, Nanjing Medical University, Nanjing 210029, China

## Abstract

Alzheimer's disease (AD) is a familiar neurodegenerative disease in the elderly. In this paper, we will review current viewpoints of microglial activation, inflammatory regulatory systems, and their relationship with AD pathology and etiology. Microglia cells are macrophage and representative of the innate immune system in brain. AD brain is marked by obvious inflammatory features, in which microglial activation is the driving force. *β*-amyloid protein sedimentation activates microglia cells, which causes the inflammation in AD. Microglia cells have dual roles: they provoke the release of inflammatory factors and cytotoxins leading to neuronal injuries and death; on the other hand, they have the neuroprotective effects. Through this, we hope to illustrate that the anti-inflammatory defenses of neurons can be practiced in the future strategy for recuperating the balance between the levels of inflammatory mediators and immune regulators in AD.

## 1. Introduction 

Alzheimer's disease (AD), also called primary Alzheimer's disease, which lives in the global the third lethal disease, is a kind of nervous system degenerative disease. Memory, judgment, thinking ability, sports and mood, feeling reaction, along with the social aging process, have become a serious threat to human life and health. The main mental derangement of this disease is characterized by nerve fibers tangles (NFT) in cells, extracellular senile plaques (SP), and a large number of neurons lost. Then, inflammation is one of the causes of Alzheimer's disease. AD may be a chronic inflammation of the central nervous system reactions, including focal brain injury and highly refractory beta-amyloid (A*β*) sedimentation; AD inflammation theory was presented. In recent years, the study found that microglia cells (MG) play a core role in the pathogenesis of AD, which is currently one of the hot topics in the study of the nervous system degenerative disease.

## 2. Microglia

Glial cells in the brain include astrocyte, oligodendrocyte, and microglia. Oligodendrocyte involves in the formation of the myelin sheath. For neurons in brain cells, astrocytes have a key role for sustaining normal and stable internal environment and tissue homeostasis. After brain damage, form and quantity of astrocyte and microglia change obviously.

As a kind of fine cell, microglia (MG) make central nervous system (CNS) perform an immune activity of scavenger sample, which is the vital element of the brain immune surveillance and the exercise of defense function in the brain. In the cells of central nervous system (CNS) they are 5~20%. Microglia originated from mesoderm bone marrow precursor cells in embryonic period, namely, the mononuclear phagocyte system. In adulthood, microglia in the brain become the innate immune cells, involved in inflammation and immune response. Kim et al. [1] found that microglia cells distribution density of the midbrain substantia nigra compacta is uneven and significantly higher than other regions; he also found that the midbrain neurons in the substantia nigra neurons are more likely to be affected than other brain area in neurodegenerative disease, which showed that the sensitive degree of nerve toxicity of the neurons may depend on the distribution density and quantity of microglia. Usually, in order to maintain the normal function of nervous system, microglia in resting state are characterized by small cell body and extend to all directions extensions of morphological characteristics. One of the most significant features of MG is very sensitive to the external environment stimulation, which is considered to be receptors in pathological cases in CNS [2]. Slight changes of central nervous system will cause microglia reaction, and the change of their forms is generally accepted as a symbol of pathology in the brain. When the brain tissue lies in the pathological state, MG are activated from the stationary state into the active state, cells body swell, synaptic disappearance, and then amoeba-like shape formation. Activate their control signals as “on” and “off”, the regulation of microglia as [3] “on” signal is a kind of new molecules, which is very obvious to identifying activated microglia; “off” signal is to maintain the signal to hamper microglia, as the default sets; the interrupt signal means the alarm sounded. The study found that the role of microglia in the development of the AD is a two-way street, that is, the immune damage and nerve protective effect, and the excessive activation can cause or aggravate neuron damage. Microglia participate in a variety of nervous system degenerative diseases and the occurrence and development of the diseases' process. In recent years, studies have shown that excessive microglial cells activation often occurred in a chronic condition, which produces a large number of cytokines and ROS production. The abnormal activation of microglia and the induced inflammation are a common link of the process which is caused by dopaminergic neuron damage.

**Figure 1:**
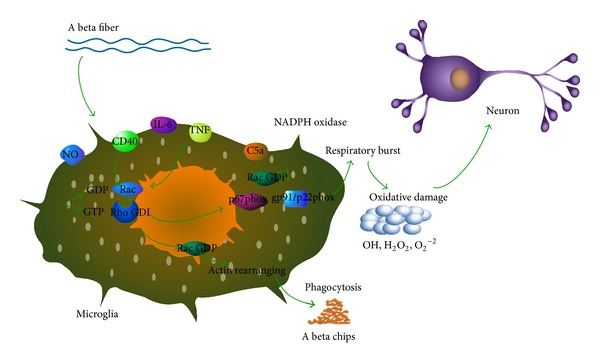
A beta activates inflammatory mediators and the complement system on microglia and then generates free radicals and makes toxic effects on neurons; on the other hand glial cells phagocytize A beta chips.

Looking from the ultrastructure, microglia into phagocytes can remove necrotic neurons and then protect the integrity of the necrotic neurons surviving around them. From the point of views of function, microglia can be activated by releasing oxygen free radical, nitric oxide, protease, and inflammatory cytokines and take the cytotoxic effect. At the same time it also can secrete nerve growth factor for supporting tissue repair [4]. Many signal molecules including many cytokines in the central nervous system of normal brain development process play an important role in neural immune regulation, which also participate in the neural pathological immune reaction in neurodegenerative diseases. These related molecules in cell surface or internal can be used as a marker of microglia and differ from the other cells in the central nervous system, such as ILB4, CD11b, and so forth. Aging and the brain A beta deposition are considered to be the major risk factors for the nerve degeneration of AD and associated with the activation of microglia (Figure 1). A beta activate microglial cells to produce a variety of inflammatory factors and make neurotoxic effect, cell inflammatory molecules which include tumor necrosis factor (TNF alpha), interleukin 1 beta (IL-1 beta), interleukin 6 (IL-6), chemokine macrophage inflammatory protein-1 (MIP-1), monocyte chemotactic protein 1 (MCP-1), complement and reactive oxygen species (ROS), and so forth [5]. These injured neurons and nerve toxic substances can cause microglia activation, which trigger a long-standing neurotoxicity cycle referred as a reactive cycle to microglial cells. Microglia activation prompts nerve injury to sustain the development for a long time, leading to reactive microglia cycle, which will exist permanently. As a result, the damage in neurons is a long-term chronic process, and it eventually leads to the development of AD. Microglia neurotransmitter receptors also express receptors, such as glutamate receptor, GABA receptors, dopamine receptor; neural hormone receptor, neuromodulation receptor, histamine receptor, cannabinoid receptor, opioid peptide receptor, chemokine receptors, TNF alpha receptors, interleukin receptors, and so forth; some recognition receptors: toll-like receptor; other receptors: formyl peptide receptor (FPR), calcium receptor, leukotriene receptor, notch receptors, and so forth. A lot of slow release of these factors may be involved in the substantia nigra striatum dopaminergic neuron degeneration necrosis of the system. A lot of studies of this view confirm that their application of dexamethasone, indomethacin, and minocycline against inflammation can protect in the model of AD dopaminergic neurons necrosis [6].

On the other hand, microglial can remove the depositing A beta by phagocytosis cells and express on the gene of antioxidant, in addition, and can secrete neurotrophic factor of nerve protection. Activated microglia can quickly increase the expression of major histocompatibility complex and then turn into antigen presenting cells and have stronger phagocytosis. Microglia express the antioxygens of heme oxygenase-1 (HO-1) and nuclear factor erythroid-related factor 2 (Nrf 2), which play a critical role in cell survival [7].

## 3. MG in Inflammatory Injury

There are many evidences that nerve cells apoptosis is closely related to the activation of MG; A beta activate MG, which mediate immune inflammatory reaction and cause the occurrence of AD which is characteristic of cells apoptosis and the cognitive decline [8]. Long-term activation of MG through the secretion of active cytokines and molecules can increase the formation of age spots and the contents of the tau protein hyperphosphorylation, which promote the nerve tangles [9]. The study indicated that N end of A beta is a necessary structure of MG to cohere; it can capture MG to age spots, and C terminal of A beta is induced by MG, which plays the role of toxic peptides. MG surface can combine with A beta receptors, such as scavenger receptors and advanced glycosylated end products receptor. Combined with A beta, microglia can be activated and produce toxic products such as free radical, cytokine, and glutamate agonists, which will accelerate neuronal death [10]. Activated MG can secrete and release TNF-alpha, IL-1, IL-6, CD40, colony stimulating factor, ring oxidase 2, prostaglandin (PG), and some chemical chemokines. These generated cytokines in turn affect the glial cells and neurons to promote the production of other inflammatory molecules for forming a positive feedback loop in the body; then inflammatory factors increase, and eventually lead to neuronal degeneration necrosis [11].

### 3.1. IL-1*β*

It is generally believed that A beta stimulate microglia to produce IL-1 beta, which can prompt the synthesis of APP and A beta deposition. IL-1 beta can increase the concentration of Ca^2+^ in neurons and cause neuron dysfunction or death. Halle et al. [12] showed that as a cytoplasm receptor, NALP3 inflammatory body is involved in regulating the activation and secretion of IL-1 beta. After being swallowed by microglia, A beta fibers activate NALP3 inflammatory body, and caspase 1, eventually, produces and releases proinflammatory cytokines IL-1 beta. A beta can activate IL-6 and IL-1; TNF alpha then releases cytokines, such as complement, chemokines, and adhesion molecules induced production increasing. These immune inflammatory factors, on the contrary, activate microglia, form a positive feedback loop in the body and produce inflammation cascade amplification effect, eventually lead to neuronal degeneration necrosis and inflammation and neurons injury in the brain [13].

### 3.2. IL-6

IL-6 is a kind of pleiotropic cytokine, it is mainly produced by the lymphocytes, fibroblasts, endothelial cells, neurons, and glial cells, which are activated by inflammation or other factors. IL-6 can prompt cute proteins to deposit in the neuritis spot and adjust the synthesis of APP. The compounds of IL-6 and sIL-r can enhance the transcription and expression of APP [14, 15]. Studies had found that IL-6 in the transgenic mouse can promote the reactive glial cell hyperplasia. At the same time, IL-1 alpha/beta, TNF alpha, intercellular adhesion molecule 1 (ICAM 1), and acute phase response proteins EB 22/5, 3 genes also raised obviously; IL-6 can further promote the neuron to degenerate in the acute phase reaction. Therefore, IL-6 plays a key role in the development of AD.

### 3.3. TNF-*α*

TNF alpha is the one of the cytokines which are secreted by the activated MG; it plays a central role in inflammatory cytokine network; its toxic effects may induce expression and secretion of a variety of the related inflammation factors through activating the cell apoptosis; it can strengthen the N-methyl-D-aspartate (N-methyl-D-aspartate, NMDA) receptor and mediate the neural toxicity and increase the glutamate which may injury the nerve cells [16]; it can also induce the colony stimulating factor (CSF) and further enlarge the inflammation reaction. In addition, it can affect the neuron membrane potential and lead to the disorder of intracellular Ca^2+^ balance for a long range, which are associated with the abnormal cell function in the process of inflammation [17, 18].

### 3.4. CD40

CD40 is a member of the TNF alpha receptor superfamily; it was found on the surface of the antigen cell like B lymphocytes, dendritic cells, and activated macrophages; its natural ligands CD40 L and CD154 express on the surface of the activated T cells [19]. The role of CD40 molecules in the proinflammatory response caused by MG has aroused the attention of many scholars, since it was found that CD40 interacts with its related ligand CD154, which leads to secrete cytokines and neurotoxic substances. Cytokines, such as interferon gamma (IFN-gamma), induce the abnormal expression of microglia by TNF alpha and facilitate the CNS neural immune cascade reaction [20]. Therefore, inhibiting the expression of CD40 can relieve the inflammatory reaction and nerve damage; eventually will be good for the treatment of nerve inflammatory diseases in the CNS.

### 3.5. Complement

Study found that A beta can activate the classic and bypass complement way; after activating the complements C3a, C4a, and C5a are produced; these small fragments have the characteristic of the inflammatory stimuli. C5a combines with the receptors on the membrane of MG and then causes a strong breathing outbreak and produces a large amounts of toxic superoxide free radicals and makes neurons damage [21]. In addition that the complement can release an amount of the inflammatory factors and cause the inflammatory reaction in the brain, it can activate MG. The inflammatory reaction can promote A beta to deposit and aggravate to form nerve plaques and then result in a vicious cycle, eventually develop AD [21].

Overall, the beneficial roles of cytokines which are produced by activated MG are very limited; the main effect is to amplify the inflammatory process and the cytotoxic effect in the different stages of AD, eventually, leading to a selective brain regions degeneration and cholinergic neuron death through the chronic and local inflammation.

## 4. The Damage of Inflammatory Medium

### 4.1. ROS

ROS is a kind of vivid oxygen free radicals which are produced in the aerobic metabolism process of cells and the material which can be converted into free radicals, mainly includes hydroxyl free radical (OH) and superoxide anion (O_2_^2−^) and hydrogen peroxide (H_2_O_2_), and so forth. ROS in the low-medium concentration play an important physiological role in body; they participate in the process of the body's immune and resist the damage to body such as exogenous bacteria and microbes. They also can be used as the second messenger to regulate and control the gene expression and the signal transduction pathway and participate in the cell growth, differentiation, and many other functions. In the condition of inflammation or trauma, too much ROS are produced and induce the cell injury and apoptosis, which is due to the excessive accumulation of the oxidation product of lipid, carbohydrate, protein and DNA, and the antioxidant system defect. ROS can be generated through various channels, but their main way is catalyzed by NADPH oxidase [22]. The NADPH oxidase is the main enzyme body, which is the origin of AD oxidative stress and is involved in the occurrence and development of AD process. The compounds of NADPH oxidase are stationary under normal circumstances; A beta deposition can make the NADPH enzyme activation. Studies have found that in AD the NADPH oxidase activity increased [23]. It indicated that it played a potential role to prompt the activation of NADPH oxidase of microglia in AD development.

### 4.2. NO

NO is an important endogenous medium of vascular activity and immune characteristics. In the central nervous system, as a special neurotransmitter NO is involved in synaptic plasticity and neuronal growth, learning and memory and behavior, and many other physiological mechanisms. But if it is released excessively, it will damage the membrane structure, protein and DNA through various channels and lead to neuronal necrosis or apoptosis [24], which eventually damage the nervous system. Nitric oxide synthase (NOS) is the unique enzyme for generating endogenous NO; it is mainly expressed by iNOS in pathological situation. Its expression is not only one of the symbols of reactive glial cell proliferation but also related to neuronal damage. INOS can continuously and catalytically catalyze to produce NO; excessive amount of NO has the potential neurotoxicity, which is an effective source of oxidative stress in the pathological process of AD.

### 4.3. Drd2

**Figure 2:**
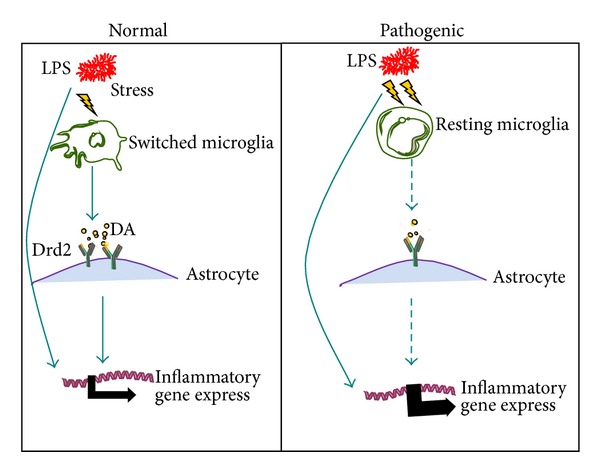
In physiological and pathological conditions, microglia transmit signal to astrocytes and astrocytes inhibit inflammation by dopamine receptors. In degenerative brain disease, microglia activation weakened, astrocytes' Drd2 signals descend too, resulting in excessive inflammation expression. DA: Dopamine.

Dopamine receptor is a vital member of the dopamine signal system of the neurotransmitter in the brain and plays a key role, including emotion, the neural activity of addiction, voluntary movement, and many other advanced functions. The studies suggest that dopamine D2 receptor (Drd2) is also expressed in microglial cells [25] and can affect the activation of CD^4+^ T lymphocyte; 18 elderly people's average level of Drd2 declined obviously [26, 27], which indicated that Drd2 may participate in the regulation of innate immunity of the central nervous system. In 2009, in the United States, Dr. Glass said that, in the inflammatory response process induced by bacterial endotoxin LPS, microglial cells were first activated, and then astrocytes accepted the immune signal of microglia and were passively activated; in both, mutual cooperation ultimately promoted the neuronal damage and degradation [28] (Figure 2). There is a kind of Cryab protein expressed in astrocytes; Cryab protein is a kind of small molecular heat shock protein, which plays the role of inhibiting inflammation and neuroprotection [29]. In the condition of outside harmful stimulations, due to the inhibitory effect of proinflammatory factor expression of Cryab, inflammation can be controlled within a certain range when the Drd2 signaling pathways of microglia to astrocytes are weakened; Cryab expression level is reduced when the outside world harmful stimulus arrives, microglia could not effectively cope with and lose effective restriction for the inflammatory response and then involve in the neuronal degeneration and aging process.

## 5. MG's Theory of Beta-Amyloid

A*β*, which is generated by the APP, plays a key role in AD. A*β* itself perhaps has no neurotoxicity; it does not cause obvious neurologic symptoms, until the inflammatory factors and A beta synergistically affect microglia and induce A beta obvious toxic effects on neuron [30]. The formation of senile plaques is due to the abnormal sedimentary of A*β*, that is, the typical pathologic characteristics of the AD. A*β*_1–40_ and A*β*_1–42_ are the most common subtypes of A*β*, which is the main component of senile plaques. They are two kinds of forms; one is the higher aggregation degree A beta [5]; the other one is a kind of smaller molecular weight of the soluble-A beta oligomers [31]. White [8] thinks that two forms of A beta have important roles in the development process of AD. Oligomers play a main role in the early stages of the disease, while A beta in the fiber form also play an important role in lasting the inflammatory response. The study found that the activated microglial cells can produce a lot of glutamic acid, which are excited by N-methyl-D-aspartic acid (NMDA) receptor through the signaling pathways, then, generating toxicity [31]. A*β* can be prompted to increase the deposition by the activated NMDA receptor outside the synapse [32]. Microglia surface has a variety of receptors combined with A*β*, such as scavenger receptor (SR) and the receptor of advanced stage glycosylation end production (RAGE) [33], which participate in the chemotaxis to microglia. In addition, a kind of soluble medium, macrophage colony-stimulating factor (M-CSF) also participated in the chemotaxis, which is activated by A*β* and secreted by microglia. The chemotactic factors, for example, MCP-1 (monocyte chemoattractant protein), prompt microglia to cluster in the deposition of A*β*; on the contrary, these chemokines of the increase recruit more microglia to which A*β* gathered [34]. Activated microglia may phagocytose A*β* through SCARA, CD36 and hydrolyze A*β* through the release of metalloproteinases, alpha secrete-enzymes, insulin hydrolytic enzymes, and so forth [35].

## 6. The Influence of Microglia in Tau Protein

Neurofibrillary tangle is one of the main pathological features of AD, under normal circumstances; the tau protein combines with tubulin, which is soluble to promote the microtubule polymerization and stability. In patients of AD, tau protein is in ana-phosphorylation; when it dissociates away from microtubules it may be from the soluble tau protein into insoluble protein, which will cause neurofibrillary tangles [36]. In animal models of tau protein, such as P301Stau transgenic mice, someone found that there gathered a large number of reactive microglia surrounding tau protein nerve cells [37]. Other studies have shown that the inflammation factor can change the activity of related kinase which caused the tau phosphorylation [38]. Sy et al. [39] found in AD transgenic mouse, the change of tau protein from soluble to insoluble was related to the inflammatory response because of the increase of the activity of GSK-3.

## 7. MG of Nerve Protective Effect

In the AD brain, MG can remove not only the necrotic and apoptosis cellular debris but also the A beta deposition. It was proved that A beta fibers in MG's cytoplasm are acquired by themselves [40]. Research has now been confirmed in the cerebral cortex of MG. It indicated that the fibers in MG are obtained by their own phagocytosis. They injected the A beta antibody in the treatment of AD and then significantly reduced the levels of A beta in the brain; for the further study of the mechanism of reducing, they found that A beta antibody can activate microglia, which then phagocytose A beta [41]. Someone queried why the activated microglia in the brain cannot effectively phagocytose A beta for the patients of AD; at present, the researchers point out that inflammatory factors are inhibiting its phagocytosis [42]. MG can also release some bioactive substances; it especially can secrete growth factors beta to support tissue repair and can limit damage and inhibit forming astrocytes scar, which have a protective effect on neurons. When brain is infected, microglia will activate and play natural immune functions, including induction of the inflammation and cytotoxic effect, and T lymphocyte antigen presenting adjustment reaction, which constitutes a defense against pathogens [43]. Activated microglia may destroy the invading microbes and remove potentially toxic cell debris and secrete growth factors to promote tissue repair; they can also synthesize a great quantity of cytokines, nutritional factors, which act as protection or injury to adjacent cells; In addition, they can also interact with other immune cells, especially with T cell in the area of inflammation in the central nervous system [44]. MG participate in the realization of the function of maintenance and repair of brain homeostasis through rapid response to physiologic and irritable stimulus. MG also play an important role for the regeneration and reconstruction of synapses, and this effect depends on the release of nerve growth factors and cytokines, and then nerve growth factor can inhibit the MG macrophage migration, local activation and help reduce the expanding lesions and inhibit the formation of new lesions.

## 8. Conclusions

More and more evidences indicate that there is a mutual promoting relationship between neuron damage and microglia activation. On the one hand, neuron damage can promote small glue toxicity to activate cells, paracrine of cytokines, and autocrine signaling; all that can lead to sustained activation of microglia. On the other hand, the chronic activation of microglia can be used as brain cytokines in the organization and continuing source of active free radicals; these factors promote together into neurons damage and form a vicious circle; it has been considered a chronic neural degeneration as an important mechanism in the process of sexual disease. The present study showed that drugs inhibiting the activation of microglia can protect dopaminergic neurons to some extent; this is also a great significance in the process of the loss of dopaminergic neurons injury from the side of microglia activation.

## 9. Expectation

The pathogenesis of AD is not yet clear at present; most scholars think the AD should be caused by a variety of reasons, and possibly in a variety of the complex pathological process of chronic ways. At present, A beta directly or indirectly affect the neurons by microglial, which is also in the debates. In a word, inflammation (immune) mechanism in AD pathological evolution may play a critical role, and NSAIDs may open up new avenues for AD prevention.
